# Supplementary material for: Pyruvate Dehydrogenase Kinase 1 inhibition mediated oxidative phosphorylation enhancement in cartilage promotes osteoarthritis progression
Source: BMC Musculoskelet Disord. 2023 Jul 20;24:597. doi: 10.1186/s12891-023-06585-6 (PMC10357736; doi:10.1186/s12891-023-06585-6)
Supplement: Supplementary file 1 — Additional file 1. [file 12891_2023_6585_MOESM1_ESM.zip › MF.Gsea.1653627316242/GOMF_STRUCTURAL_CONSTITUENT_OF_RIBOSOME.html]

Details for gene set GOMF\_STRUCTURAL\_CONSTITUENT\_OF\_RIBOSOME[GSEA]

|  || Dataset | 55457\_collapsed\_to\_symbols.cls.cls #OA\_versus\_normal.cls.cls #OA\_versus\_normal\_repos |
| Phenotype | cls.cls#OA\_versus\_normal\_repos |
| Upregulated in class | normal |
| GeneSet | GOMF\_STRUCTURAL\_CONSTITUENT\_OF\_RIBOSOME |
| Enrichment Score (ES) | -0.42068595 |
| Normalized Enrichment Score (NES) | -1.9240319 |
| Nominal p-value | 0.0 |
| FDR q-value | 0.032115538 |
| FWER p-Value | 0.162 |
Table: GSEA Results Summary

  

Fig 1: Enrichment plot: GOMF\_STRUCTURAL\_CONSTITUENT\_OF\_RIBOSOME      
 Profile of the Running ES Score & Positions of GeneSet Members on the Rank Ordered List

  

| SYMBOL | TITLE | RANK IN GENE LIST | RANK METRIC SCORE | RUNNING ES | CORE ENRICHMENT || 1 | MRPL16 | mitochondrial ribosomal protein L16 [Source:HGNC Symbol;Acc:HGNC:14476] | 132 | 0.874 | 0.0070 | No |
| 2 | MRPS14 | mitochondrial ribosomal protein S14 [Source:HGNC Symbol;Acc:HGNC:14049] | 207 | 0.814 | 0.0173 | No |
| 3 | MRPL18 | mitochondrial ribosomal protein L18 [Source:HGNC Symbol;Acc:HGNC:14477] | 289 | 0.757 | 0.0260 | No |
| 4 | MRPL49 | mitochondrial ribosomal protein L49 [Source:HGNC Symbol;Acc:HGNC:1176] | 292 | 0.755 | 0.0408 | No |
| 5 | MRPL57 | mitochondrial ribosomal protein L57 [Source:HGNC Symbol;Acc:HGNC:14514] | 314 | 0.740 | 0.0538 | No |
| 6 | MRPS33 | mitochondrial ribosomal protein S33 [Source:HGNC Symbol;Acc:HGNC:16634] | 513 | 0.655 | 0.0513 | No |
| 7 | MRPL34 | mitochondrial ribosomal protein L34 [Source:HGNC Symbol;Acc:HGNC:14488] | 884 | 0.547 | 0.0332 | No |
| 8 | MRPS16 | mitochondrial ribosomal protein S16 [Source:HGNC Symbol;Acc:HGNC:14048] | 994 | 0.519 | 0.0350 | No |
| 9 | MRPL11 | mitochondrial ribosomal protein L11 [Source:HGNC Symbol;Acc:HGNC:14042] | 1028 | 0.512 | 0.0425 | No |
| 10 | MRPL35 | mitochondrial ribosomal protein L35 [Source:HGNC Symbol;Acc:HGNC:14489] | 1034 | 0.510 | 0.0522 | No |
| 11 | NDUFA7 | NADH:ubiquinone oxidoreductase subunit A7 [Source:HGNC Symbol;Acc:HGNC:7691] | 1097 | 0.496 | 0.0572 | No |
| 12 | MRPS11 | mitochondrial ribosomal protein S11 [Source:HGNC Symbol;Acc:HGNC:14050] | 1305 | 0.457 | 0.0501 | No |
| 13 | MRPL22 | mitochondrial ribosomal protein L22 [Source:HGNC Symbol;Acc:HGNC:14480] | 1311 | 0.456 | 0.0587 | No |
| 14 | MRPL15 | mitochondrial ribosomal protein L15 [Source:HGNC Symbol;Acc:HGNC:14054] | 1479 | 0.432 | 0.0542 | No |
| 15 | MRPS7 | mitochondrial ribosomal protein S7 [Source:HGNC Symbol;Acc:HGNC:14499] | 1650 | 0.406 | 0.0490 | No |
| 16 | MRPL13 | mitochondrial ribosomal protein L13 [Source:HGNC Symbol;Acc:HGNC:14278] | 1655 | 0.406 | 0.0567 | No |
| 17 | MRPL17 | mitochondrial ribosomal protein L17 [Source:HGNC Symbol;Acc:HGNC:14053] | 1714 | 0.396 | 0.0600 | No |
| 18 | MRPL24 | mitochondrial ribosomal protein L24 [Source:HGNC Symbol;Acc:HGNC:14037] | 1843 | 0.379 | 0.0575 | No |
| 19 | DAP3 | death associated protein 3 [Source:HGNC Symbol;Acc:HGNC:2673] | 2028 | 0.358 | 0.0502 | No |
| 20 | MRPL42 | mitochondrial ribosomal protein L42 [Source:HGNC Symbol;Acc:HGNC:14493] | 2198 | 0.339 | 0.0437 | No |
| 21 | MRPL23 | mitochondrial ribosomal protein L23 [Source:HGNC Symbol;Acc:HGNC:10322] | 2210 | 0.337 | 0.0495 | No |
| 22 | MRPL4 | mitochondrial ribosomal protein L4 [Source:HGNC Symbol;Acc:HGNC:14276] | 2247 | 0.333 | 0.0533 | No |
| 23 | RPL36AL | ribosomal protein L36a like [Source:HGNC Symbol;Acc:HGNC:10346] | 2255 | 0.332 | 0.0593 | No |
| 24 | MRPS18A | mitochondrial ribosomal protein S18A [Source:HGNC Symbol;Acc:HGNC:14515] | 2295 | 0.327 | 0.0628 | No |
| 25 | SRBD1 | S1 RNA binding domain 1 [Source:HGNC Symbol;Acc:HGNC:25521] | 2571 | 0.294 | 0.0471 | No |
| 26 | ISG15 | ISG15 ubiquitin like modifier [Source:HGNC Symbol;Acc:HGNC:4053] | 2821 | 0.268 | 0.0329 | No |
| 27 | RPS10P5 | ribosomal protein S10 pseudogene 5 [Source:HGNC Symbol;Acc:HGNC:15795] | 2879 | 0.262 | 0.0336 | No |
| 28 | MRPL44 | mitochondrial ribosomal protein L44 [Source:HGNC Symbol;Acc:HGNC:16650] | 3306 | 0.220 | 0.0046 | No |
| 29 | MRPS22 | mitochondrial ribosomal protein S22 [Source:HGNC Symbol;Acc:HGNC:14508] | 3331 | 0.218 | 0.0071 | No |
| 30 | RPL36A | ribosomal protein L36a [Source:HGNC Symbol;Acc:HGNC:10359] | 3387 | 0.213 | 0.0070 | No |
| 31 | MRPS15 | mitochondrial ribosomal protein S15 [Source:HGNC Symbol;Acc:HGNC:14504] | 3475 | 0.205 | 0.0043 | No |
| 32 | MRPS30 | mitochondrial ribosomal protein S30 [Source:HGNC Symbol;Acc:HGNC:8769] | 3645 | 0.190 | -0.0052 | No |
| 33 | RPL18 | ribosomal protein L18 [Source:HGNC Symbol;Acc:HGNC:10310] | 3749 | 0.182 | -0.0097 | No |
| 34 | MRPS31 | mitochondrial ribosomal protein S31 [Source:HGNC Symbol;Acc:HGNC:16632] | 3844 | 0.171 | -0.0136 | No |
| 35 | RPS27L | ribosomal protein S27 like [Source:HGNC Symbol;Acc:HGNC:18476] | 3882 | 0.166 | -0.0132 | No |
| 36 | RPL18A | ribosomal protein L18a [Source:HGNC Symbol;Acc:HGNC:10311] | 4049 | 0.151 | -0.0232 | No |
| 37 | RPL39L | ribosomal protein L39 like [Source:HGNC Symbol;Acc:HGNC:17094] | 4075 | 0.149 | -0.0222 | No |
| 38 | MRPL33 | mitochondrial ribosomal protein L33 [Source:HGNC Symbol;Acc:HGNC:14487] | 4241 | 0.133 | -0.0325 | No |
| 39 | RPL26L1 | ribosomal protein L26 like 1 [Source:HGNC Symbol;Acc:HGNC:17050] | 4584 | 0.106 | -0.0572 | No |
| 40 | MRPS17 | mitochondrial ribosomal protein S17 [Source:HGNC Symbol;Acc:HGNC:14047] | 4898 | 0.080 | -0.0801 | No |
| 41 | RPL10A | ribosomal protein L10a [Source:HGNC Symbol;Acc:HGNC:10299] | 4974 | 0.074 | -0.0845 | No |
| 42 | UBA52 | ubiquitin A-52 residue ribosomal protein fusion product 1 [Source:HGNC Symbol;Acc:HGNC:12458] | 4978 | 0.074 | -0.0833 | No |
| 43 | RPS26 | ribosomal protein S26 [Source:HGNC Symbol;Acc:HGNC:10414] | 5368 | 0.043 | -0.1129 | No |
| 44 | MRPL12 | mitochondrial ribosomal protein L12 [Source:HGNC Symbol;Acc:HGNC:10378] | 5396 | 0.041 | -0.1142 | No |
| 45 | MRPS35 | mitochondrial ribosomal protein S35 [Source:HGNC Symbol;Acc:HGNC:16635] | 5425 | 0.039 | -0.1156 | No |
| 46 | RPS19 | ribosomal protein S19 [Source:HGNC Symbol;Acc:HGNC:10402] | 5952 | -0.002 | -0.1568 | No |
| 47 | MRPL9 | mitochondrial ribosomal protein L9 [Source:HGNC Symbol;Acc:HGNC:14277] | 5962 | -0.002 | -0.1574 | No |
| 48 | MRPL20 | mitochondrial ribosomal protein L20 [Source:HGNC Symbol;Acc:HGNC:14478] | 5989 | -0.004 | -0.1594 | No |
| 49 | RSL24D1 | ribosomal L24 domain containing 1 [Source:HGNC Symbol;Acc:HGNC:18479] | 5992 | -0.004 | -0.1595 | No |
| 50 | MRPS18C | mitochondrial ribosomal protein S18C [Source:HGNC Symbol;Acc:HGNC:16633] | 6145 | -0.016 | -0.1711 | No |
| 51 | MRPL19 | mitochondrial ribosomal protein L19 [Source:HGNC Symbol;Acc:HGNC:14052] | 6172 | -0.018 | -0.1728 | No |
| 52 | RPL15 | ribosomal protein L15 [Source:HGNC Symbol;Acc:HGNC:10306] | 6185 | -0.019 | -0.1733 | No |
| 53 | RPL35A | ribosomal protein L35a [Source:HGNC Symbol;Acc:HGNC:10345] | 6383 | -0.034 | -0.1881 | No |
| 54 | RPL10L | ribosomal protein L10 like [Source:HGNC Symbol;Acc:HGNC:17976] | 6502 | -0.042 | -0.1965 | No |
| 55 | RPL8 | ribosomal protein L8 [Source:HGNC Symbol;Acc:HGNC:10368] | 6606 | -0.050 | -0.2035 | No |
| 56 | MRPL3 | mitochondrial ribosomal protein L3 [Source:HGNC Symbol;Acc:HGNC:10379] | 6811 | -0.064 | -0.2182 | No |
| 57 | RPL12 | ribosomal protein L12 [Source:HGNC Symbol;Acc:HGNC:10302] | 6845 | -0.067 | -0.2195 | No |
| 58 | RPL36 | ribosomal protein L36 [Source:HGNC Symbol;Acc:HGNC:13631] | 6860 | -0.068 | -0.2193 | No |
| 59 | RPS27A | ribosomal protein S27a [Source:HGNC Symbol;Acc:HGNC:10417] | 6973 | -0.078 | -0.2265 | No |
| 60 | MRPL46 | mitochondrial ribosomal protein L46 [Source:HGNC Symbol;Acc:HGNC:1192] | 7326 | -0.104 | -0.2520 | No |
| 61 | MRPS12 | mitochondrial ribosomal protein S12 [Source:HGNC Symbol;Acc:HGNC:10380] | 7328 | -0.104 | -0.2500 | No |
| 62 | RPL35 | ribosomal protein L35 [Source:HGNC Symbol;Acc:HGNC:10344] | 7563 | -0.124 | -0.2658 | No |
| 63 | MRPS2 | mitochondrial ribosomal protein S2 [Source:HGNC Symbol;Acc:HGNC:14495] | 7863 | -0.146 | -0.2864 | No |
| 64 | RPL3L | ribosomal protein L3 like [Source:HGNC Symbol;Acc:HGNC:10351] | 8097 | -0.163 | -0.3014 | No |
| 65 | MRPS18B | mitochondrial ribosomal protein S18B [Source:HGNC Symbol;Acc:HGNC:14516] | 8145 | -0.166 | -0.3018 | No |
| 66 | RPS21 | ribosomal protein S21 [Source:HGNC Symbol;Acc:HGNC:10409] | 8394 | -0.185 | -0.3175 | No |
| 67 | RPS9 | ribosomal protein S9 [Source:HGNC Symbol;Acc:HGNC:10442] | 8508 | -0.194 | -0.3225 | No |
| 68 | RPL29 | ribosomal protein L29 [Source:HGNC Symbol;Acc:HGNC:10331] | 8661 | -0.205 | -0.3303 | No |
| 69 | MRPL28 | mitochondrial ribosomal protein L28 [Source:HGNC Symbol;Acc:HGNC:14484] | 8841 | -0.221 | -0.3400 | No |
| 70 | RPSA | ribosomal protein SA [Source:HGNC Symbol;Acc:HGNC:6502] | 8866 | -0.222 | -0.3375 | No |
| 71 | RPL28 | ribosomal protein L28 [Source:HGNC Symbol;Acc:HGNC:10330] | 8985 | -0.230 | -0.3421 | No |
| 72 | RPLP2 | ribosomal protein lateral stalk subunit P2 [Source:HGNC Symbol;Acc:HGNC:10377] | 9185 | -0.246 | -0.3528 | No |
| 73 | RPL37 | ribosomal protein L37 [Source:HGNC Symbol;Acc:HGNC:10347] | 9265 | -0.253 | -0.3540 | No |
| 74 | RPS25 | ribosomal protein S25 [Source:HGNC Symbol;Acc:HGNC:10413] | 9282 | -0.254 | -0.3502 | No |
| 75 | RPL4 | ribosomal protein L4 [Source:HGNC Symbol;Acc:HGNC:10353] | 9770 | -0.298 | -0.3824 | No |
| 76 | RPL5 | ribosomal protein L5 [Source:HGNC Symbol;Acc:HGNC:10360] | 10201 | -0.341 | -0.4093 | No |
| 77 | RPL24 | ribosomal protein L24 [Source:HGNC Symbol;Acc:HGNC:10325] | 10263 | -0.347 | -0.4072 | No |
| 78 | RPL22 | ribosomal protein L22 [Source:HGNC Symbol;Acc:HGNC:10315] | 10270 | -0.348 | -0.4008 | No |
| 79 | RPS8 | ribosomal protein S8 [Source:HGNC Symbol;Acc:HGNC:10441] | 10525 | -0.376 | -0.4132 | Yes |
| 80 | RPL31 | ribosomal protein L31 [Source:HGNC Symbol;Acc:HGNC:10334] | 10533 | -0.377 | -0.4063 | Yes |
| 81 | RPS14 | ribosomal protein S14 [Source:HGNC Symbol;Acc:HGNC:10387] | 10558 | -0.379 | -0.4007 | Yes |
| 82 | RPS7 | ribosomal protein S7 [Source:HGNC Symbol;Acc:HGNC:10440] | 10608 | -0.384 | -0.3969 | Yes |
| 83 | RPL10 | ribosomal protein L10 [Source:HGNC Symbol;Acc:HGNC:10298] | 10684 | -0.391 | -0.3950 | Yes |
| 84 | RPS11 | ribosomal protein S11 [Source:HGNC Symbol;Acc:HGNC:10384] | 10734 | -0.397 | -0.3910 | Yes |
| 85 | RPL17 | ribosomal protein L17 [Source:HGNC Symbol;Acc:HGNC:10307] | 10806 | -0.406 | -0.3885 | Yes |
| 86 | RPL7A | ribosomal protein L7a [Source:HGNC Symbol;Acc:HGNC:10364] | 10811 | -0.406 | -0.3807 | Yes |
| 87 | RPS4X | ribosomal protein S4 X-linked [Source:HGNC Symbol;Acc:HGNC:10424] | 10812 | -0.406 | -0.3727 | Yes |
| 88 | RPS29 | ribosomal protein S29 [Source:HGNC Symbol;Acc:HGNC:10419] | 10913 | -0.419 | -0.3722 | Yes |
| 89 | RPS4Y1 | ribosomal protein S4 Y-linked 1 [Source:HGNC Symbol;Acc:HGNC:10425] | 10932 | -0.422 | -0.3652 | Yes |
| 90 | RPL13 | ribosomal protein L13 [Source:HGNC Symbol;Acc:HGNC:10303] | 11013 | -0.431 | -0.3629 | Yes |
| 91 | RPL38 | ribosomal protein L38 [Source:HGNC Symbol;Acc:HGNC:10349] | 11122 | -0.446 | -0.3626 | Yes |
| 92 | RPL6 | ribosomal protein L6 [Source:HGNC Symbol;Acc:HGNC:10362] | 11129 | -0.447 | -0.3542 | Yes |
| 93 | RPL14 | ribosomal protein L14 [Source:HGNC Symbol;Acc:HGNC:10305] | 11154 | -0.451 | -0.3471 | Yes |
| 94 | RPS5 | ribosomal protein S5 [Source:HGNC Symbol;Acc:HGNC:10426] | 11206 | -0.458 | -0.3420 | Yes |
| 95 | RPS3 | ribosomal protein S3 [Source:HGNC Symbol;Acc:HGNC:10420] | 11245 | -0.463 | -0.3358 | Yes |
| 96 | RPL11 | ribosomal protein L11 [Source:HGNC Symbol;Acc:HGNC:10301] | 11252 | -0.464 | -0.3271 | Yes |
| 97 | RPS10 | ribosomal protein S10 [Source:HGNC Symbol;Acc:HGNC:10383] | 11408 | -0.488 | -0.3295 | Yes |
| 98 | RPL32 | ribosomal protein L32 [Source:HGNC Symbol;Acc:HGNC:10336] | 11447 | -0.496 | -0.3227 | Yes |
| 99 | RPS20 | ribosomal protein S20 [Source:HGNC Symbol;Acc:HGNC:10405] | 11507 | -0.502 | -0.3174 | Yes |
| 100 | RPL21 | ribosomal protein L21 [Source:HGNC Symbol;Acc:HGNC:10313] | 11647 | -0.524 | -0.3179 | Yes |
| 101 | RPS6 | ribosomal protein S6 [Source:HGNC Symbol;Acc:HGNC:10429] | 11662 | -0.526 | -0.3085 | Yes |
| 102 | RPS15 | ribosomal protein S15 [Source:HGNC Symbol;Acc:HGNC:10388] | 11671 | -0.527 | -0.2987 | Yes |
| 103 | RPS16 | ribosomal protein S16 [Source:HGNC Symbol;Acc:HGNC:10396] | 11755 | -0.541 | -0.2944 | Yes |
| 104 | RPL27 | ribosomal protein L27 [Source:HGNC Symbol;Acc:HGNC:10328] | 11765 | -0.544 | -0.2844 | Yes |
| 105 | RPL19 | ribosomal protein L19 [Source:HGNC Symbol;Acc:HGNC:10312] | 11967 | -0.587 | -0.2885 | Yes |
| 106 | RPS27 | ribosomal protein S27 [Source:HGNC Symbol;Acc:HGNC:10416] | 12071 | -0.612 | -0.2844 | Yes |
| 107 | RPS17 | ribosomal protein S17 [Source:HGNC Symbol;Acc:HGNC:10397] | 12130 | -0.626 | -0.2765 | Yes |
| 108 | RPL30 | ribosomal protein L30 [Source:HGNC Symbol;Acc:HGNC:10333] | 12140 | -0.629 | -0.2648 | Yes |
| 109 | RPS12 | ribosomal protein S12 [Source:HGNC Symbol;Acc:HGNC:10385] | 12156 | -0.632 | -0.2534 | Yes |
| 110 | RPL13A | ribosomal protein L13a [Source:HGNC Symbol;Acc:HGNC:10304] | 12200 | -0.645 | -0.2440 | Yes |
| 111 | RPS24 | ribosomal protein S24 [Source:HGNC Symbol;Acc:HGNC:10411] | 12223 | -0.652 | -0.2328 | Yes |
| 112 | RPLP0 | ribosomal protein lateral stalk subunit P0 [Source:HGNC Symbol;Acc:HGNC:10371] | 12285 | -0.672 | -0.2242 | Yes |
| 113 | RPS15A | ribosomal protein S15a [Source:HGNC Symbol;Acc:HGNC:10389] | 12291 | -0.674 | -0.2113 | Yes |
| 114 | RPS13 | ribosomal protein S13 [Source:HGNC Symbol;Acc:HGNC:10386] | 12334 | -0.687 | -0.2009 | Yes |
| 115 | RPL3 | ribosomal protein L3 [Source:HGNC Symbol;Acc:HGNC:10332] | 12350 | -0.691 | -0.1884 | Yes |
| 116 | RPLP1 | ribosomal protein lateral stalk subunit P1 [Source:HGNC Symbol;Acc:HGNC:10372] | 12467 | -0.734 | -0.1829 | Yes |
| 117 | RPL7 | ribosomal protein L7 [Source:HGNC Symbol;Acc:HGNC:10363] | 12519 | -0.757 | -0.1719 | Yes |
| 118 | RPS28 | ribosomal protein S28 [Source:HGNC Symbol;Acc:HGNC:10418] | 12526 | -0.761 | -0.1573 | Yes |
| 119 | RPS2 | ribosomal protein S2 [Source:HGNC Symbol;Acc:HGNC:10404] | 12541 | -0.769 | -0.1432 | Yes |
| 120 | RPL23 | ribosomal protein L23 [Source:HGNC Symbol;Acc:HGNC:10316] | 12558 | -0.777 | -0.1290 | Yes |
| 121 | RPL27A | ribosomal protein L27a [Source:HGNC Symbol;Acc:HGNC:10329] | 12592 | -0.792 | -0.1159 | Yes |
| 122 | RPL34 | ribosomal protein L34 [Source:HGNC Symbol;Acc:HGNC:10340] | 12602 | -0.799 | -0.1008 | Yes |
| 123 | RPS18 | ribosomal protein S18 [Source:HGNC Symbol;Acc:HGNC:10401] | 12611 | -0.804 | -0.0854 | Yes |
| 124 | RPS23 | ribosomal protein S23 [Source:HGNC Symbol;Acc:HGNC:10410] | 12661 | -0.834 | -0.0727 | Yes |
| 125 | RPL41 | ribosomal protein L41 [Source:HGNC Symbol;Acc:HGNC:10354] | 12674 | -0.847 | -0.0569 | Yes |
| 126 | RPL37A | ribosomal protein L37a [Source:HGNC Symbol;Acc:HGNC:10348] | 12754 | -0.918 | -0.0449 | Yes |
| 127 | RPS3A | ribosomal protein S3A [Source:HGNC Symbol;Acc:HGNC:10421] | 12758 | -0.921 | -0.0269 | Yes |
| 128 | RPL23A | ribosomal protein L23a [Source:HGNC Symbol;Acc:HGNC:10317] | 12785 | -0.944 | -0.0102 | Yes |
| 129 | RPL39 | ribosomal protein L39 [Source:HGNC Symbol;Acc:HGNC:10350] | 12797 | -0.963 | 0.0081 | Yes |
Table: GSEA details [plain text format]

  

Fig 2: GOMF\_STRUCTURAL\_CONSTITUENT\_OF\_RIBOSOME      
 Blue-Pink O' Gram in the Space of the Analyzed GeneSet

  

Fig 3: GOMF\_STRUCTURAL\_CONSTITUENT\_OF\_RIBOSOME: Random ES distribution      
 Gene set null distribution of ES for **GOMF\_STRUCTURAL\_CONSTITUENT\_OF\_RIBOSOME**
